# Early Onset Primary Hyperparathyroidism Associated with a Novel Germline Mutation in *CDKN1B*

**DOI:** 10.1155/2015/510985

**Published:** 2015-07-14

**Authors:** Marianne S. Elston, Goswin Y. Meyer-Rochow, Michael Dray, Michael Swarbrick, John V. Conaglen

**Affiliations:** ^1^Department of Endocrinology, Waikato Hospital, Private Bag 3200, Hamilton 3240, New Zealand; ^2^Faculty of Medicine and Health Sciences, University of Auckland, Waikato Clinical Campus, Private Bag 3200, Hamilton 3240, New Zealand; ^3^Department of Surgery, Waikato Hospital, Private Bag 3200, Hamilton 3240, New Zealand; ^4^Department of Pathology, Waikato Hospital, Private Bag 3200, Hamilton 3240, New Zealand; ^5^Department of Radiology, Waikato Hospital, Private Bag 3200, Hamilton 3240, New Zealand

## Abstract

Individuals presenting with primary hyperparathyroidism (PHPT) at a young age commonly have an underlying germline gene mutation in one of the following genes: *MEN1, CASR*, or *CDC73*. A small number of families with primary hyperparathyroidism have been identified with germline mutations in *CDKN1B* and those patients with primary hyperparathyroidism have almost exclusively been women who present in middle age suggesting that the age of onset of PHPT in MEN4 may be later than that of MEN1. We present a case of apparently sporadic PHPT presenting in adolescence with single gland disease associated with a novel *CDKN1B* germline mutation (heterozygote for a missense mutation in exon 1 of the *CDKN1B* gene (c.378G>C) (p.E126D)). The implication from this case is that *CDKN1B* germline mutations may be associated with PHPT at an earlier age than previously thought.

## 1. Introduction

Primary hyperparathyroidism (PHPT) is a relatively common endocrine disorder with a prevalence of approximately 6.7/1000 in the adult population [1]. PHPT is more common in women and is typically diagnosed between the ages of 40 and 75 years [1, 2]. Most cases of PHPT are sporadic, that is, not familial or part of a syndrome. Primary hyperparathyroidism presenting at a young age is uncommon and needs to be differentiated from familial hypocalciuric hypercalcaemia (FHH) in order to avoid unnecessary surgery for the latter disorder. Young patients with PHPT are more likely to have an underlying germline mutation in genes such as* MEN1*,* CASR*,* RET*, or* CDC73* [3]. Germline mutations in cyclin-dependent kinase inhibitor 1B (*CDKN1B*) have also recently been identified to be associated with a Multiple Endocrine Neoplasia (MEN) syndrome which may include PHPT, termed MEN4 (OMIM number 610755) [4]. In MEN1 syndrome, tumours occur in the parathyroid glands, pancreas, and pituitary and less commonly elsewhere, whereas in the very rare MEN4 syndrome a broader spectrum of organ pathology including PHPT, pituitary, and pancreatic tumours occurs, with neuroendocrine tumours also described at a variety of other sites (e.g., cervix, bronchus, and stomach) [5–9].* CDKN1B* encodes p27(kip1), a cyclin-dependent kinase 2 inhibitor involved with the control of the cell cycle at G1 [10]. To date, only a small number of families have been identified with germline mutations in* CDKN1B* and of those cohorts, patients with primary hyperparathyroidism have almost exclusively been women who present in middle age [4–9, 11, 12]. This suggests that the age of onset of PHPT in MEN4 may be later than that of MEN1 [5]. The questions are raised as to at what age screening for PHPT in* CDKN1B* carriers should start and whether assessment of germline mutations in* CDKN1B* should be considered in patients with familial or young onset PHPT in the absence of other gene mutations predisposing to PHPT.

We report a case of primary hyperparathyroidism presenting with renal stones at age 15 associated with a novel germline heterozygous missense mutation in* CDKN1B*.

## 2. Case Presentation

A previously fit and well 15-year-old girl presented with recurrent renal calculi. Her serum calcium was elevated at 2.9 mmol/L (reference range (RR) 2.1–2.55 mmol/L), with a reduced phosphate of 0.63 mmol/L (RR 0.7–1.5 mmol/L) and increased PTH at 11.6 pmol/L (RR 1.3–6.8 pmol/L). Twenty-four-hour urine calcium was elevated at 16.3 mmol/d (RR 2.5–7.5 mmol/d) and urine calcium/creatinine clearance ratio was 0.025, consistent with primary hyperparathyroidism. There was no clinical or biochemical evidence of any other endocrinopathy. There was no family history of calcium or other endocrine disorders and both of her parents had a normal serum calcium level.

Initial sestamibi imaging suggested an enlarged left lower parathyroid gland but at surgery only brown fat was present in this location and four-gland exploration failed to identify a parathyroid adenoma or the left inferior parathyroid. The other parathyroid glands appeared normal. Additional imaging studies, including 4D CT scanning, repeat sestamibi, and MRI, were inconclusive. Selective venous sampling demonstrated markedly elevated levels of PTH in the thymic vein consistent with an adenoma in the anterior mediastinum. This was unable to be removed by a cervical approach and was resected via an upper hemisternotomy resulting in normalization of her calcium and parathyroid hormone status. Histology demonstrated a parathyroid adenoma surrounded by normal thymic parenchyma. Parafibromin staining of the adenoma was positive and PGP9.5 negative making a* CDC73* germline mutation unlikely.

**Figure 1:**
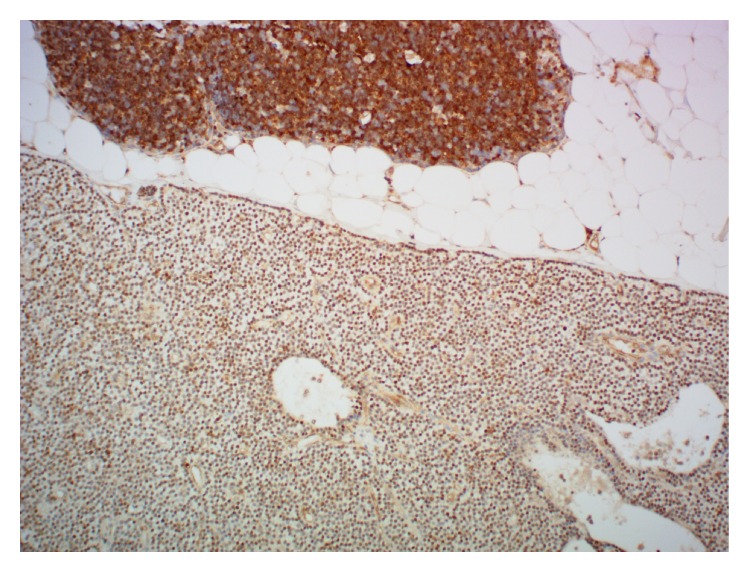
Immunohistochemical staining for p27 (original magnification ×100). Positive nuclear staining for parathyroid adenoma in lower half and positive nuclear staining for thymic lymphocytes in upper half.

Despite the lack of a family history of calcium disorders, given her young age genetic testing was offered to determine whether there was a predisposing germline mutation. PCR and Sanger sequencing of leukocyte DNA for the* MEN1*,* CASR* (all coding regions and flanking intronic regions), and* RET* (exons 10–16 including splice junctions) genes did not identify a mutation. However she was found to be heterozygote for a novel missense mutation in exon 1 of the* CDKN1B* gene (c.378G>C) resulting in an amino acid substitution (p.E126D). Her mother (aged 46) and maternal grandfather (aged 74 years) also carried the same missense mutation but both were normocalcemic with normal PTH levels. The immunohistochemical staining pattern of the adenoma for p27 (mouse monoclonal antibody clone SX53G8, Cell Marque) demonstrated normal nuclear staining (Figure 1).

## 3. Discussion

We present a case of apparently sporadic PHPT presenting in adolescence with single gland disease and a novel* CDKN1B* germline mutation.

Primary hyperparathyroidism is rare in children and adolescents. In a Scottish epidemiological study in which adults aged 20+ years with probable or definite PHPT were identified from community biochemistry data, based on an elevated serum calcium level with inappropriately normal or elevated PTH levels, only 1.4% of PHPT occurred in patients under the age of 30 years [1]. In that study, patients aged <20 years were excluded because of the increased likelihood of FHH; however, FHH was not excluded in those patients aged between 20 and 30 years [1]. As not all patients in that study had confirmed PHPT based on detailed clinical assessment and surgical outcomes, the true rate of definite PHPT is likely to be less than the 1.4% reported. In a large American study of surgically treated patients with PHPT only 0.86% (88/10190) of patients were aged <20 years [2]. A higher rate of 2.1% of patients <20 years with PHPT (21/1000) was found in another US study of surgically treated PHPT but the authors reported that this proportion was probably falsely increased due to referral bias [13]. As such, it is likely that if patients with FHH are excluded, <2% of cases of PHPT occur in patients <30 years. Interestingly, young patients who do develop PHPT are reported to be more liable to be symptomatic than their adult counterparts, that is, more likely to have renal stones, fatigue, depression, and weakness [13].

PHPT may be sporadic (accounting for the vast majority of cases) or occur as part of a familial syndrome associated with a germline mutation in one of the predisposing genes such as* MEN1*,* RET*,* CASR*,* CDC73*, or* CDKN1B*. Young onset PHPT has been reported to be more frequently associated with an underlying germline mutation [3]. In a recent study patients who had undergone parathyroid surgery and were aged <45 years at the time of surgery were offered germline mutation testing of* MEN1*,* RET*,* CDC73*, and* CASR* [3]. Of the 102 patients, 16 patients had familial PHPT identified either preoperatively or as part of the work-up (11 MEN1, 4 MEN2a, and 1 HPT-JT). Of the remaining 86 patients who had nonsyndromic, apparently sporadic disease 8 further patients were identified as having relevant germline mutations (4* MEN1*, 3* CASR*, and 1* CDC73*). Overall, in that cohort of patients aged <45 years at the time of surgery, 24% of those tested had an underlying germline mutation identified [3]. Conversely, in a study from a different unit of surgically treated young PHPT patients aged <20 years, 18/21 had single gland disease and of the 3 patients with multigland disease only 1 was identified as having an underlying germline mutation (*MEN1*) [13]. However, it is unclear from this paper as to whether all patients underwent germline testing and which genes were assessed [13]. In a Northern Finnish cohort where testing for* MEN1*,* CDC73*,* CASR*,* CDKN1B*, and* AIP* genes was offered to all patients presenting with PHPT aged <40 years or with multigland or recurrent disease or family history of PHPT or MEN1 only 1/29 had a mutation detected (which was in* MEN1*) [14].

Guidelines from the Proceedings of the Fourth International Workshop on Asymptomatic PHPT recommend testing those who present at a young age (although the age is not specified), the presence of multigland disease, parathyroid carcinoma, or atypical adenoma, and those with a family history or evidence of syndromic disease [15]. In the absence of syndromic features the recommended guidelines for sequence of gene testing based on order of likely frequency are* MEN1*,* CASR*,* AP2S1*,* GNA11*,* CDC73*, and* CDKN1A/1B/2C* genes,* RET*, and* PTH* [15]. Screening is useful in identifying carriers who can continue to be monitored for both PHPT and other tumours associated with the syndrome for which they carry a gene mutation. Investigating family members for specific gene mutations also allows those who do not carry the germline mutation to be reassured and avoids the expense of ongoing assessment and testing of these individuals.

In our unit, due to financial constraint, in the absence of familial or syndromic features we offer genetic testing only to those with an onset of PHPT aged <30 years (*MEN1*,* RET*,* CDKN1B*,* CASR*, and* CDC73* (with germline mutation testing for the latter being guided by parafibromin staining of the parathyroid lesion [16])). Until more clinical data is available to suggest a different approach, we also limit testing for* AP2S1* and* GNA11* to those with an FHH phenotype. This practice is likely to miss some cases in which the penetrance is low or if a detailed family history is not available.

Germline* CDKN1B* mutations are associated with MEN4 [4]. To date, only a few families have been reported [4–9, 11, 12]. Based on the limited published data, the penetrance of primary hyperparathyroidism in MEN4 is assumed to be fairly high, although there remains a lack of large well-documented families to confirm this concept. Interestingly, the diagnosis of primary hyperparathyroidism in MEN4 appears to occur later than MEN1 and predominantly in women with the average age reported being 56 years compared to 20–25 years for MEN1 [5]. In our case, we have identified early onset primary hyperparathyroidism, with its associated complications (renal stones) present at age 15 associated with a germline* CDKN1B* variant but no other features of MEN4. If the E126D* CDKN1B* variant is pathogenic, it would suggest that* CDKN1B* mutations may need to be considered in young patients presenting with PHPT when other mutations are not identified. Detailed assessment of the family we describe has not yet revealed any other endocrinopathies in affected members or their untested relatives suggesting that the disease penetrance for this particular missense mutation may be low.

The missense mutation identified in the proband (c.378G>C) has not previously been reported. The base change results in an amino acid substitution of glutamic acid to aspartic acid at position 126 (E126D) in exon 1. The region in which this change occurs is a highly conserved area in a c-Jun activation domain-binding protein-1 (JAB1) binding domain. JAB1 is important in shuttling p27 from the nucleus to the cytoplasm and in p27 degradation [17]. PolyPhen-2, a predictive software tool which predicts the possible impact of an amino acid substitution on the structure and function of a human gene [18], predicts the E126D variant as probably damaging (score 0.996, sensitivity 0.55, and specificity 0.98). In this case normal p27 immunohistochemical staining was present in the parathyroid adenoma.* CDKN1B* is somewhat atypical for a tumour suppressor gene as, rather than two “hits” being necessary for the development of disease [19], haploinsufficiency is thought to potentially be sufficient [20]. A limitation of this paper is that functional studies have not been performed to confirm that this variant is pathological; however despite extensive testing no other cause for the patient's PHPT was identified making it possible that this germline variant was a predisposing factor.

## 4. Conclusions

Young onset PHPT (aged <30 years) is uncommon and even in the absence of syndromic features and multigland disease an underlying germline mutation in one of the PHPT predisposing genes should be considered. This case suggests that* CDKN1B* mutations may be associated with early onset PHPT.
